# Health Information Brokers in the General Population: An Analysis of the Health Information National Trends Survey 2013-2014

**DOI:** 10.2196/jmir.5447

**Published:** 2016-06-03

**Authors:** Sarah L Cutrona, Kathleen M Mazor, Amenah A Agunwamba, Sruthi Valluri, Patrick M Wilson, Rajani S Sadasivam, Lila J Finney Rutten

**Affiliations:** ^1^ University of Massachusetts Medical School Department of Medicine Worcester, MA United States; ^2^ Meyers Primary Care Institute Worcester, MA United States; ^3^ University of Massachusetts Medical School Department of Quantitative Health Sciences Worcester, MA United States; ^4^ Robert D. and Patricia E. Kern Center for the Science of Healthcare Delivery Mayo Clinic Rochester, MN United States; ^5^ University of Minnesota Medical School Minneapolis, MN United States; ^6^ University of Minnesota School of Public Health Department of Epidemiology and Community Health Minneapolis, MN United States

**Keywords:** health information seeking, peer communication, social network, patient self-management, health care decision-making

## Abstract

**Background:**

Health information exchanged between friends or family members can influence decision making, both for routine health questions and for serious health issues. A health information broker is a person to whom friends and family turn for advice or information on health-related topics. Characteristics and online behaviors of health information brokers have not previously been studied in a national population.

**Objective:**

The objective of this study was to examine sociodemographic characteristics, health information seeking behaviors, and other online behaviors among health information brokers.

**Methods:**

Data from the Health Information National Trends Survey (2013-2014; n=3142) were used to compare brokers with nonbrokers. Modified Poisson regression was used to examine the relationship between broker status and sociodemographics and online information seeking.

**Results:**

Over half (54.8%) of the respondents were consulted by family or friends for advice or information on health topics (ie, they acted as health information brokers). Brokers represented 54.1% of respondents earning <$20,000 yearly and 56.5% of respondents born outside the United States. Women were more likely to be brokers (PR 1.34, 95% CI 1.23-1.47) as were those with education past high school (PR 1.42, CI 1.22-1.65). People aged ≥75 were less likely to be brokers as compared to respondents aged 35-49 (PR 0.81, CI 0.67-0.99). Brokers used the Internet more frequently for a variety of online behaviors such as seeking health information, creating and sharing online content, and downloading health information onto a mobile device; and also reported greater confidence in obtaining health information online.

**Conclusions:**

More than 50% of adults who responded to this national survey, including those with low income and those born abroad, were providing health information or advice to friends and family. These individuals may prove to be effective targets for initiatives supporting patient engagement and disease management, and may also be well-positioned within their respective social networks to propagate health messages.

## Introduction

Health information exchanged between friends or family members influences decision making, both for routine health questions and for serious health concerns [1-5]. In 2014, 60% of Americans reported that they obtained health information or support from friends and family for a difficult health issue [5]. While health care professionals remain the preferred source of information for many technical questions, family and friends offer factual health information and also emotional support, drawing on personal experiences, beliefs, and attitudes [3,5]. Recognizing and supporting those who provide health care advice to their peers (both online and offline) may be an effective way to disseminate health messages to broader audiences.

Various terms have been used to describe the roles played by laypeople while providing health information to family or friends. Studies have described surrogate seekers (those who self-report seeking health information on behalf of someone other than themselves) and lay information mediaries (nonprofessionals who seek information on behalf of others without necessarily being asked to do so); related concepts include “gatekeeping,” “proxy information seeking,” “sharing information found for others on the Web,” “information-acquiring and information-sharing,” or completion of an “imposed query” or “gift query” [6-11].

These terms have been used to convey slightly different meanings, but all emphasize the act of seeking and acquiring health information before passing it on. Previous literature has described use of family, friends, or other lay interpersonal contacts as sources of health information for a broad range of topics [3,12]. Interpersonal sources of health information tend to be female, in good or excellent health, living in shared household arrangements (marriage, living with others, providing care to an adult relative) and tend to be related to someone with a serious or chronic medical condition [11,13,14]. These individuals may engage in online activities requiring user-generated content (eg, email communication with health care providers, participation in online health support groups) and often work to help patients overcome information-seeking barriers [14,15]. Those comfortable with online communication may act as intermediaries for members of at-risk populations who lack the ability (due to language, literacy, cognitive challenges, or ease with technology) to search and access online information, bridging the “digital divide” that has been described among traditionally disadvantaged groups [16]. Many factors may motivate a person to seek health information on behalf of others. This activity can stem from feelings of empathy, altruism, and a desire to help; helping behavior may also be pleasurable and thus benefit the helper [10,15,17].

Health information acquired through personal experience can also be of instrinsic value to social network members, whether passed along verbally or organized and made more accessible through the Internet [1]. Previous research defines individuals with knowledge on a health-related subject (and who consider it important to share this knowledge with others) as “health information mavens” [18]. Among low-income Massachusetts respondents, mavenism has been associated with certain characteristics (being female, older, with larger social networks and with moderate consumption of general media) [18]. In one study, mavens had also spent fewer years in the United States and had lower language acculturation levels [18]. While this study provides valuable information about a selected group of individuals, these findings may not be generalizable to a broader population. Further studies are needed to better characterize those persons who act as sources of health information for friends and family.

Using data from the Health Information National Trends Survey (HINTS) [19], we identified respondents who were acting as brokers of health information. A “health information broker” is a person to whom friends and family turn for information or advice on health related topics. We sought to describe sociodemographic characteristics, health information seeking behaviors (or lack thereof), and online health information communication preferences among brokers compared with nonbrokers. In order to understand whether our findings were applicable among traditionally disadvantaged groups, we additionally studied characteristics of brokers among respondents with low incomes and among those born outside the United States. Understanding characteristics of health information brokers may have implications related to the design of health communication campaigns, including future eHealth and mHealth interventions targeting these users.

## Methods

### Data Collection and Response Rates

Data for these analyses were obtained from the HINTS, a national survey of the US adult population that assesses knowledge, attitudes, and behavior related to health communication and related outcomes [19]. HINTS 4, Cycle 3 is the only version of HINTS fielded thus far to specifically ask whether “family members and friends ask you for information or advice on health topics”. Data for HINTS 4, Cycle 3 were collected from September 6, 2013 to December 30, 2013 (*n*=12,010) through mailed questionnaires. The sample design for HINTS 4, Cycle 3 was a two-stage, stratified sample, wherein addresses were selected from a comprehensive national residential file from the United States Postal Service, and individual respondents were selected for each sampled household. The final response rate was 35.19% (*n*=3142) [20]. Details on sampling strategies and survey design are available in the HINTS 4, Cycle 3 methodology report [21]. HINTS 4 was approved by the Westat Institutional Review Board (IRB) in an expedited review in 2010, and was deemed exempt from IRB review by the National Institutes of Health (NIH) Office of Human Subjects Research in 2011.

### Measures

#### Sociodemographic Variables

The following sociodemographic variables were included in these analyses: sex (male, female), age (18-34, 35-49, 50-64, 65-74, and 75+ years), race or ethnicity (non-Hispanic white, non-Hispanic black, Hispanic or Latino, and non-Hispanic “other”), annual household income (< $20K, $20 to <$35K, $35K to <$50, $50K to <$75K, $75K or more), education (less than high school or high school graduate, some college, college/Bachelor’s degree), born in the United States (yes, no), speaks English (not at all or not well, well, very well), and marital status (married or living as married, not married).

#### Health Information Broker Status

Each respondent was asked “Do family members and friends ask you for information or advice on health topics?” Those who responded affirmatively were classified as health information brokers.

#### Health Information Seeking and Sources

To assess health information seeking behavior, respondents were asked “Have you ever looked for information about health or medical topics from any source?” Those who answered “yes” were asked “For whom was the information sought during the most recent search (myself, someone else, both)?” and “The most recent time you looked for information about health or medical topics, where did you go first?” Responses were coded as family or friends, health care provider, Internet, print materials, and other sources.

On a 4-point scale, ranging from “Not at all” to “A lot,” respondents were asked to rate the extent to which they trusted information about health or medical topics from the following 8 sources: doctor, family or friends, news, radio, Internet, television, government health agencies, and charitable /religious organizations. The question did not address the language in which this information was delivered. Responses were dichotomized into a lot versus all other responses. For news sources, a respondent who indicated that they had ‘a lot’ of trust in online newspapers, print newspapers, special health magazines, medical magazines or newsletters was categorized as having ‘a lot’ of trust in information from news sources; for television, a respondent who indicated that they had ‘a lot’ of trust in local or national television was categorized as having ‘a lot’ of trust in information from television.

#### Information Seeking Experiences

All respondents were asked to rate their degree of confidence in their ability to obtain necessary health or medical information on a scale ranging from “completely confident” to “not confident at all.” For this analysis, responses were dichotomized as: completely or very confident and somewhat, a little, or not at all confident.

Each respondent was asked about their information source preferences with the following question: “Imagine that you had a strong need to get information about health or medical topics. Where would you go first?” Responses were coded as printed materials, family or friends, Internet, healthcare provider, and “other.”

#### Internet Use

Patients were questioned on their use of the Internet; those who indicated that they use the Internet were asked about their online activities including social networking site visits, sharing health information on social networking sites, writing in an online diary or blog, participating in an online support group related to health issues, and viewing health-related YouTube videos. Online activities in the last 12 months were also documented, which included seeking health or medical information on behalf of oneself or others, seeking information on smoking cessation, purchasing of medicine or vitamins online, seeking a health care provider online, downloading health information to a mobile device, tracking personal health information, and communicating online with a doctor or with personnel in the doctor’s office.

#### Data Analyses

Analysis of the complex survey data was conducted using SAS 9.3 and the survey package for R version 3.02 [22,23]. All data were weighted to provide estimates of the US population and to correct for nonresponse bias, and all standard errors were calculated using replicate weights. For the analysis of health information brokering, we conducted cross-tabulation and chi-square statistics to evaluate the relationship between brokering and sociodemographic characteristics, and between brokering and online information seeking experiences. Additionally, we performed cross-tabulation and chi-square statistics on the following subgroups: those with income <$20,000 (*n*=744) and those born outside the United States (*n*=508), again examining the relationship between brokering and sociodemographic characteristics and online information seeking behaviors and experiences. All chi-square statistics had a Rao–Scott correction to account for the complex nature of the survey. A multivariable modified Poisson regression analysis was conducted examining the independent associations of age, sex, race or ethnicity, household income (imputed), and education with health information brokering. Modified Poisson regression was used because in cases of high prevalence, the prevalence ratio (PR) is a better approximation to the relative risk than the odds ratio [24]. Curators of HINTS data use Cox-Iannacchione weighted sequential hot deck imputation to impute values for missing income data.

## Results

Our final sample included 3142 respondents. Approximately half (54.8 **%**) of the respondents reported acting as health information brokers.

### Sociodemographic Characteristics of Brokers (Brokers vs Nonbrokers)

On bivariate analyses (Table 1), brokers were more frequently female (58.4% of brokers were women vs 43.8% of nonbrokers; *P*<.001). Brokers reported higher incomes (36.0% earned ≥ $75,000 per year vs 29.0% of nonbrokers; *P*=.02) and higher educational levels (73.6% had at least some college degree vs 56.8% of nonbrokers; *P*<.001). Respondents between the ages 35 and 64 acted as brokers most frequently (32.7% of health information brokers were aged 35-49 vs 27.7% of nonbrokers; 26.5% of brokers were aged 50-64 vs 23.5% of nonbrokers, *P*=.01). Compared to nonbrokers, a higher percentage of brokers were married (61.6% of brokers vs 55.1% of nonbrokers; *P*=.036).

**Table 1:** Sociodemographic characteristics of health information brokers^a^

Characteristic		Total	Health information broker	Not health information broker	*P* value^c^
		*N* (%)^b^	*N* (%)^b^	*N* (%)^b^	
Overall		3142 (100)	1774 (54.8)	1368 (45.2)	
Age					.013
	18-34	422 (27.22)	239 (25.97)	183 (28.74)	
	35-49	701 (30.39)	428 (32.65)	273 (27.65)	
	50-64	1060 (25.13)	628 (26.49)	432 (23.49)	
	65-74	504 (9.36)	275 (8.80)	229 (10.04)	
	75+	355 (7.89)	150 (6.09)	205 (10.08)	
Sex					<.001
	Female	1882 (51.77)	1179 (58.37)	703 (43.78)	
	Male	1179 (48.23)	549 (41.63)	630 (56.22)	
Education					<.001
	Less than/High school	977 (33.96)	471 (26.37)	506 (43.18)	
	Some college	924 (32.79)	531 (36.63)	393 (28.12)	
	College	1155 (33.25)	728 (37.00)	427 (28.70)	
Race/Ethnicity					.990
	White, non-Hispanic	1576 (67.27)	859 (67.37)	717 (67.15)	
	Black, non-Hispanic	416 (10.44)	258 (10.35)	158 (10.55)	
	Hispanic	491 (15.02)	291 (14.81)	200 (15.28)	
	Other, non-Hispanic	208 (7.27)	125 (7.48)	83 (7.02)	
Household income					.019
	Less than $20,000	744 (20.75)	402 (20.34)	342 (21.26)	
	$20,000 to < $35,000	437 (13.99)	226 (11.20)	211 (17.42)	
	$35,000 to < $50,000	430 (14.52)	238 (13.82)	192 (15.37)	
	$50,000 to < $75,000	495 (17.87)	285 (18.64)	210 (16.92)	
	$75,000 or more	880 (32.88)	538 (36.00)	342 (29.03)	
Currently employed					.733
	Employed	1600 (61.61)	936 (62.05)	664 (61.08)	
	Not employed	1468 (38.39)	800 (37.95)	668 (38.92)	
Marital status					.036
	Divorced/Separated	603 (11.02)	340 (11.33)	263 (10.63)	
	Married, living as married	1572 (58.68)	936 (61.61)	636 (55.10)	
	Never married	529 (24.52)	288 (22.40)	241 (27.11)	
	Widowed	343 (5.78)	161 (4.66)	182 (7.16)	
Born in the United States					.624
	Yes	2582 (83.95)	1444 (83.44)	1138 (84.57)	
	No	508 (16.05)	303 (16.56)	205 (15.43)	
Speaks English					.067
	Very well	2578 (87.41)	1486 (89.42)	1092 (84.97)	
	Well	269 (7.65)	129 (6.18)	140 (9.44)	
	Not at all, not well	160 (4.94)	94 (4.40)	66 (5.59)	

^a^Health information brokers: respondents to the Health Information National Trends Survey (HINTS 2013-2014) who answer yes to the question: “Do family members and friends ask you for information or advice on health topics?

^b^Percentages are weighted according to the US population estimates in the American Community Survey to provide representative estimates of the adult US population.

^c^Rao–Scott chi-square test, missing excluded

### Brokers’ Health Information Seeking Experiences

On bivariate analyses (Table 2), brokers more frequently reported having looked for information on health or medical topics from any source (86.2% of brokers had sought health information vs 67.4% of nonbrokers, *P*<.001). Brokers also expressed confidence in their own ability to find information on health and medical topics as needed (58.8% of brokers vs 51.9% of nonbrokers indicated that they felt completely or very confident; *P*=.02). When participants were asked to rate their trust in information about health or medical topics, brokers and nonbrokers did not significantly differ in trust of information derived from the Internet. Brokers less frequently reported trusting their doctor “a lot” compared to nonbrokers (66.1% of brokers vs 72.0% of nonbrokers; *P*=.01).

Brokers more frequently engaged in health information seeking on behalf of others. Almost half of the brokers (48.1%) reported that their most recent information search was on behalf of someone else as compared to 33.8% of non-brokers (*P*<.001).

A lower percentage of brokers reported that they would first consult a health care provider (46.6% of brokers vs 54.8% of nonbrokers; *P*=.021) while many brokers cited the Internet as their first resource for health information when required (44.5% of brokers would use Internet first vs 36.4% of nonbrokers; *P*=.021). While not all surrogate searches were Internet-based, brokers more frequently reported use of the Internet for surrogate health information seeking in the previous 12 months (79.2% of brokers had used the Internet to seek medical information on behalf of someone else vs 52.8% of nonbrokers; *P*<.001 (see Table 3).

### Internet Experience

Health information brokers demonstrated greater use of the Internet (84.4% of brokers had used the Internet vs 71.4% of nonbrokers; *P*<.001). Once online, brokers more frequently pursued a number of health information seeking activities. Compared to nonbrokers, a higher proportion of brokers used the Internet to look for a health care provider, to download health information, and to track personal health information (Table 3). Health-related YouTube videos had been viewed by 42.3% of brokers as compared to only 25.2% of nonbrokers (*P*<.001). While both brokers and nonbrokers visited social networking sites (77.4% of brokers vs 74.3% of nonbrokers; *P* =NS ), brokers shared health information on such sites more frequently (30.0% of brokers vs 14.5% of nonbrokers; *P*<.001). Brokers frequently participated in an online forums or support groups for people with similar health or medical issues (8.6% of brokers vs 4.7% of nonbrokers; *P*=.034) and communicated online with a doctor or someone in the doctor’s office (36.3% of brokers vs 20.4% of nonbrokers; *P*<.001).

### Low-Income Health Information Brokers

Among respondents earning less than $20,000 annually (*n*=744), 54.1% were health information brokers. Education level was the only demographic variable associated with health information brokering among low-income respondents; compared to those with high school or less education, those with greater than high school education more frequently acted as brokers (53.4% of brokers vs 38.9% of nonbrokers; *P*=.0138). No other demographic variables were significantly associated with brokering among those with annual incomes less than $20,000.

**Figure 1:**
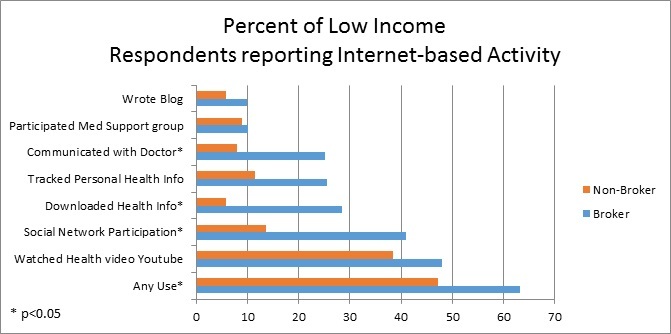
Comparison of health information brokers versus nonbrokers within low-income respondents (<$20,000 annually; n=744).

Additionally, among those with low incomes, rates of seeking health information (from any source) were higher for brokers (81.1% vs 56.3% of nonbrokers; *P*<.001) and brokers were more often engaged in online surrogate seeking (72.6% vs 44.1%; *P*=.007). Low-income brokers more frequently reported trust in the Internet (15.8% had ‘a lot’ of trust in information from the Internet vs. 5.0% of non-brokers; *P*=.02). Brokers in this low-income group (compared to nonbrokers) used the Internet more often (see Figure 1) and were more likely to have participated in a social networking site, downloaded health information and used the Internet to communicate with doctors or personnel in doctors’ offices. We also observed a trend toward more frequent online tracking of personal health information among brokers (25.5% of brokers vs 11.6%; *P*=.06).

**Table 2:** Health information seeking experiences of health information brokers^a^

Experience	Total	Health information Broker	Not health information Broker		*P*-value^c^
	*N* (%)^b^	*N* (%)^b^	*N* (%)^b^		
*Ever looked for health information (any source)?*					<.001					
Yes	2482 (77.72)	1528 (86.20)	954 (67.43)		
No	660 (22.28)	246 (13.80)	414 (32.57)		
*Most recent searched, sought health information for whom?*					<.001	
Self	1466 (57.51)	820 (51.92)	646 (66.22)		
Someone else	409 (18.70)	272 (20.28)	137 (16.26)		
Both self and someone else	587 (23.78)	426 (27.80)	161 (17.52)		
*Confidence in ability to obtain needed health information*					.020	
Complete/Very	1779 (55.72)	1059 (58.81)	720 (51.90)		
Somewhat/A little/Not at all	1292 (44.28)	688 (41.19)	604 (48.10)		
*Where did you seek health information most recently?*					.362	
Family/Friends	92 (4.12)	56 (4.18)	36 (4.03)		
Health care provider	400 (14.77)	229 (13.03)	171 (17.55)		
Internet	1328 (69.50)	824 (71.37)	504 (66.53)		
Print material	241 (9.46)	144 (9.18)	97 (9.91)		
Other	62 (2.14)	39 (2.24)	23 (1.97)		
*If strongly needed, where would you seek health information?*					.021	
Family/Friends	136 (4.37)	65 (3.80)	71 (5.07)		
Health care provider	1625 (50.25)	863 (46.55)	762 (54.75)		
Internet	1043 (40.82)	647 (44.46)	396 (36.39)		
Print material	99 (2.69)	51 (3.10)	48 (2.18)		
Other	69 (1.87)	39 (2.09)	30 (1.61)		
*Trust information on health or medical topics from doctor*					.014	
A lot	2135 (68.77)	1188 (66.12)	947 (71.99)		
Some/A little/Not at all	961 (31.23)	564 (33.88)	397 (28.01)		
*Trust information on health or medical topics from family or friends*					.185	
A lot	230 (8.40)	140 (7.20)	90 (9.86)		
Some/A little/Not at all	2773 (91.60)	1570 (92.80)	1203 (90.14)		
*Trust information on health or medical topics from news*					.204	
A lot	688 (24.15)	418 (25.50)	270 (22.51)		
Some/A little/Not at all	2329 (75.85)	1300 (74.50)	1029 (77.49)		
*Trust information on health or medical topics from radio*					.056	
A lot	68 (1.74)	35 (1.11)	33 (2.51)		
Some/A little/Not at all	2883 (98.26)	1642 (98.89)	1241 (97.49)		
*Trust information on health or medical topics from the Internet*					.056	
A lot	417 (12.31)	268 (14.04)	149 (10.18)		
Some/A little/Not at all	2530 (87.69)	1412 (85.96)	1118 (89.82)		
*Trust information on health or medical topics from television*					.551	
A lot	237 (7.88)	148 (7.35)	89 (8.52)		
Some/A little/Not at all	2771 (92.12)	1564 (92.65)	1207 (91.48)		
*Trust information on health or medical topics from government*					.424	
A lot	765 (26.58)	462 (27.54)	303 (25.41)		
All others	2204 (73.42)	1230 (72.46)	974 (74.59)		
*Trust information on health or medical topics from charities or religious organizations*					.367	
A lot	296 (9.46)	185 (10.14)	111 (8.63)		
All others	2700 (90.54)	1523 (89.86)	1177 (91.37)		

^a^Health information brokers: respondents to the Health Information National Trends Survey (HINTS 2013-2014) who answer yes to the question: “Do family members and friends ask you for information or advice on health topics?

^b^Percentages are weighted according to the US population estimates in the American Community Survey to provide representative estimates of the adult US population.

^c^Rao–Scott chi-square test, missing excluded

### Health Information Brokers Born Outside the United States

Subgroup analyses restricted to respondents born outside of the United States (*n*=508) show that 56.5% of them were brokers. As with low-income brokers, education was the only sociodemographic variable significantly associated with broker status among foreign-born respondents (69.5 % of brokers vs 47.1% of nonbrokers had completed education past high school; *P*=.0012).

**Figure 2:**
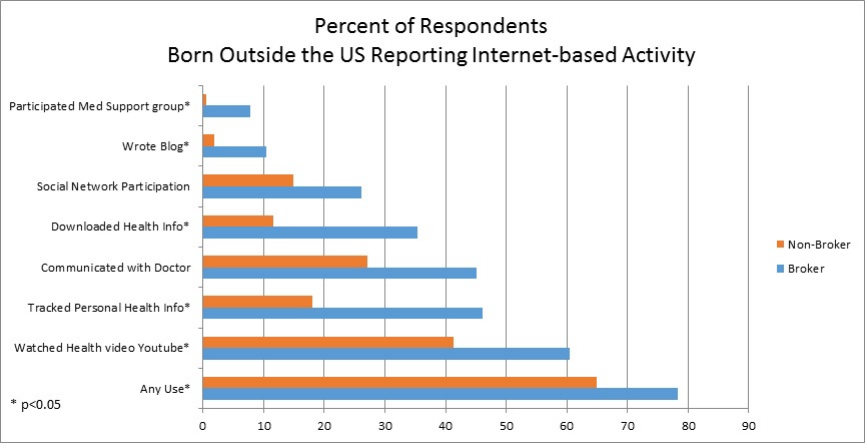
Comparison of health information brokers versus nonbrokers among respondents born outside the United States (n=508).

Similar to the overall population and to the low-income subgroup, rates of seeking health information (from any source) were higher for foreign-born brokers than nonbrokers (81.9% vs 51.5% respectively; *P*<.001) and foreign-born brokers more often sought health information on the Internet for someone else (81.5% vs 50.2%; *P*<.001). Foreign-born brokers showed no significant difference in trusting the Internet but less often trusted health or medical information from television (10.4% vs 25.8% of foreign-born nonbrokers; *P*=.007) or from charities or religious organizations (10.7% vs 24.1%; *P*=.004); they selected the Internet as a preferred source of information more frequently (45.6% vs 31.1% of nonbrokers; *P*=.001). As shown in Figure 2 , a higher proportion of these brokers reported some form of Internet use. Compared to foreign born nonbrokers, these brokers were more likely to have written in an online diary or blog, participated in an online medical support group, watched a health video on YouTube, downloaded health information, and tracked personal health information online. We found a trend toward increased frequency of Internet-based communication with a personnel in the doctor’s office (45.1% of brokers vs 27.1% of nonbrokers; *P*=.09).

**Table 3:** Internet experiences and online health information exchange preferences expressed by health information brokers^a^

Experience	Total	Health information broker	Not health information broker		*P* -value^c^
	*N* (%)^b^	*N* (%)^b^	*N* (%)^b^		
*Used Internet*					<.001	
Yes	2266 (78.51)	1393 (84.36)	873 (71.39)		
No	858 (21.49)	373 (15.64)	485 (28.61)		
*Visited social networking site such as Facebook or LinkedIn* ^d^					.334	
Yes	1625 (76.14)	1012 (77.41)	613 (74.33)		
No	628 (23.86)	369 (22.59)	259 (25.67)		
*Shared health information on social networking site such as Facebook or Twitter* ^d^					<.001	
Yes	446 (23.64)	333 (30.02)	113 (14.53)		
No	1803 (76.36)	1045 (69.98)	758 (85.47)		
*Wrote in online diary or blog* ^d^					.176	
Yes	138 (6.35)	91 (7.24)	47 (5.06)		
No	2109 (93.65)	1286 (92.76)	823 (94.94)		
*Participated in online forum or support group for people with similar health or medical issues* ^d^					.034	
Yes	150 (7.02)	115 (8.61)	35 (4.74)		
No	2097 (92.98)	1263 (91.39)	834 (95.26)		
*Watched health-related video on YouTube* ^d^					<.001	
Yes	702 (35.30)	508 (42.34)	194 (25.23)		
No	1543 (64.70)	867 (57.66)	676 (74.77)		
*Used the Internet to*					
*Look for medical information for yourself* ^d^					<.001	
Yes	1808 (79.66)	1175 (85.41)	633 (71.47)		
No	439 (20.34)	200 (14.59)	239 (28.53)		
*Look for medical information for someone else* ^d^					<.001	
Yes	1465 (68.28)	1038 (79.15)	427 (52.75)		
No	775 (31.72)	333 (20.85)	442 (47.25)		
*Look for information on quitting smoking*					.284	
Yes	159 (9.13)	108 (8.27)	51 (10.37)		
No	2079 (90.87)	1262 (91.73)	817 (89.63)		
*Buy medicine or vitamins*					.007	
Yes	456 (20.30)	302 (23.18)	154 (16.18)		
No	1784 (79.70)	1070 (76.82)	714 (83.82)		
*Look for a healthcare provider*					.004	
Yes	826 (38.85)	560 (43.40)	266 (32.36)		
No	1407 (61.15)	803 (56.60)	604 (67.64)		
*Download health information to mobile device*					<.001	
Yes	432 (20.02)	341 (27.43)	91 (9.43)		
No	1807 (79.98)	1031 (72.57)	776 (90.57)		
*Track personal health information (eg, care received, test results, medical appointments)*					.001	
Yes	698 (28.29)	497 (33.64)	201 (20.66)		
No	1541 (71.71)	873 (66.36)	668 (79.34)		
*Communicate with doctor or personnel in doctor’s office*					<.001	
Yes	666 (29.74)	458 (36.32)	208 (20.35)		
No	1576 (70.26)	914 (63.68)	662 (79.65)		

^a^Health information brokers: respondents to the Health Information National Trends Survey (2013-2014) who answered yes to the question: “Do family members and friends ask you for information or advice on health topics?"

^b^Percentages are weighted according to the US population estimates in the American Community Survey to provide representative estimates of the adult US population.

^c^Rao–Scott chi-square test, missing excluded

^d^In the last 12 months

### Multivariable Models

Multivariable models based on the entire sample (Table 4) predicting broker status from age, sex, race or ethnicity, income level, and educational level found that those whose education was greater than high school were significantly more likely to have acted as brokers (PR 1.42, 95% CI, 1.22-1.65) compared to those with education of high school or less. Women were significantly more likely to report acting as brokers compared to men (PR 1.34, 95% CI, 1.23-1.47) and elderly respondents aged 75 and above were significantly less likely to report acting as brokers when compared with those aged 35-49 years (PR 0.81, 95% CI, 0.67-0.99).

**Table 4:** Multivariate analysis.

Characteristics		Predicting information broker status	
		Prevalence ratio (PR)	95% CI
Age (35-49)			
	18-34	0.85	0.72-1.01
	50-64	0.94	0.84-1.07
	65-74	0.90	0.78-1.05
	75+	0.81^a^	0.67-0.99
Female		1.34^b^	1.23-1.47
Race (White, non-Hispanic)			
	Black, non-Hispanic	0.99	0.84-1.17
	Asian, non-Hispanic	0.99	0.75-1.30
	Hispanic	1.13	0.94-1.35
	Other/Unknown	1.05	0.86-1.28
Household income imputed ($75,000 or more)			
	Less than $20,000	0.97	0.80-1.18
	$20,000-< $35,000	0.82	0.65-1.04
	$35,000-< $50,000	0.92	0.79-1.07
	$50,000-< $75,000	1.01	0.87-1.17
Education (≤high school)			
	Above high school	1.42^b^	1.22-1.65
*Observations*		2870	

^a^ *P*<.05

^b^ *P*<.001

## Discussion

### Principal Findings

To our knowledge, this is the first study to examine characteristics and online behaviors of health information brokers within a national population. Our study builds on previous research, highlighting the widespread nature of health information brokering and documenting characteristics of brokers. As a group, health information brokers were more often female and had higher educational levels. Although higher rates of brokering were seen amongst those in the highest income groups, we found this behavior to be present across all income levels. We also found comparable brokering rates among those born in and those born outside the United States. Our findings add to the literature by providing a deeper understanding of health information brokers’ online behavior. Brokers used the Internet more often for a variety of tasks associated with health information-seeking and information exchange. They also sought information on behalf of others more frequently and reported greater confidence in their own ability to obtain needed health information. Finally, this study offers insight into the existence of brokering activity among those who do not view themselves as active seekers of information. While the majority of brokers described themselves as health information seekers, more than 1 out of 10 brokers did not. This subset of brokers merits further investigation to understand whether they view themselves as sources of health information or advice based on their own experiences as opposed to their skill in information seeking.

We found over half of the respondents had engaged in health information brokering activity; this documentation of the widespread use of interpersonal sources of health information is consistent with previous studies [5,25]. According to a 2014 Pew study, when faced with a serious health issue, the majority of adults surveyed (60%) turned to friends and family for health information or support; one-quarter of those surveyed (24%) sought information or support from patients with the same health condition and 13% of adults conducted online searches to find others with health concerns similar to theirs, a practice more common among those with chronic and rare medical conditions [5]. In a survey assessing cancer screening decision-making among a randomly selected national sample of adults 50 years and above [2], authors found that family and friends were frequently ranked as an extremely important source of information for prostate, colorectal, and breast cancer screening, second only to health care providers. Studies in the US estimate that 56-66% of those responding to national surveys have sought health information on behalf of others [13,14]. In contrast, a 2011 online survey administered to online health information seekers in the United Kingdom who were accessing the National Health Service direct website showed that only 30% sought information for others [26].

Individuals possessing knowledge on a health topic (whether derived from searches or personal experience) and who consider it important to share this information with others have been described previously as “health information mavens.” Kontos et al [18] found that being female was associated with scoring higher on a 5-item scale measuring “health information mavenism”; similarly, we found that health information brokers were more likely to be female. Our study adds to the current understanding of health information brokering activity, documenting an association between higher education and brokering. The relation between education and scores was not directly examined by Kontos et al, but participants had both a lower frequency of education past the high school level (7% compared with 76% in our study) and an overall lower percentage of respondents self-identifying as mavens (44%). Women and those with higher education have been identified previously as more likely to engage in seeking health information on behalf of others [13,14].

While we did not find a significant relationship between brokering and either place of birth or language in our national sample, Kontos et al found that those living in the United States for less than 10 years had slightly higher scores than others. In addition, their study found that those who did not speak English as a primary language but read and spoke English occasionally at home were more likely to have higher scores than those who had English as their primary language. For more recent immigrants and for those with developing English skills, Kontos et al postulated that being part of an interdependent community might have a protective effect, adding that the ability to “effectively communicate in both English and their native language” likely made these individuals “conduits of information, including health information, for their community” [18].

Our understanding of the online behaviors of health information brokers indicates that they are more active than the nonbrokers in online communities. Our study shows that these individuals are more likely to create and exchange online content, whether by participating in social networks and medical support groups or by communicating with personnel in doctors’ offices, and they are more likely to seek, download, and track health information. Similar online behaviors have been identified previously among those who seek health information on behalf of others [13,14].

While the “digital divide” has narrowed in recent years, there remain disparities in Internet use and access. These disparities disproportionately affect the elderly and those who are less affluent, have less education or who live in rural parts of the country [27,28]. Brokers may help decrease the impact of this divide by accessing and passing along health information; however, more research is needed to understand the quality of the information disseminated by brokers. Commonly used search engines such as Google do not retrieve health information based on the quality (or appropriateness for a given health literacy level) and information shared through online communities and support groups is not always accurate. This places the onus on the broker to judge the quality and relevance of the information before sharing it with others. Additional insights into the attitudes attitudes and behaviors of brokers may be useful for improving Internet-based dissemination of high-quality health information.

Based on our findings, further exploration of ways in which brokers support patient engagement and disease management is encouraged. Our findings indicate that brokers are more likely to have communicated online with doctors or personnel in the doctor’s office than nonbrokers, but it is not clear whether brokers are doing this on behalf of themselves or for someone else (for instance, through secure messaging via proxy portal access within an electronic health record, EHR). Future studies might examine the current use and potential benefits incurred by granting brokers (with permission from patients) proxy access to patient EHRs. Such access may help brokers who are already partnering with patients in acute illness and in chronic disease self-management efforts. Family or friends who accompany patients during healthcare visits could also be directed to approved online resources such as the Centers for Disease Control and Prevention (CDC) caregiving resources (http://www.cdc.gov/aging/caregiving/resources.htm). This can be accomplished via hyperlinks in the patient portal and could facilitate access to quality information on behalf of patients in the ambulatory setting, and (where available) on behalf of hospitalized patients (via applications such as Epic MyChart Bedside). Additional functions on technology-based interventions could be developed to support proxy access and communication of information to others. Family or friends could also be provided information on recommended websites via printed care summaries, which are routinely provided to patients at the end of ambulatory visits.

Studies examining the link between social network characteristics and broker behavior would help us understand whether health communication campaigns would benefit from targeting health information brokers. Such studies could also examine whether being a health information broker is a static characteristic or a behavior which changes over time.

Finally, our study highlights the need for further investigation into brokering among those who do not view themselves as active seekers of information; in particular, brokers who dispense information and advice based on personal experience with the medical system.

### Limitations

Some limitations of this research are worth noting. HINTS data are cross-sectional; therefore causality cannot be inferred. Response rates for HINTS, while consistent with other national surveys, were low [29]. National surveys, such as HINTS, are very often constrained by survey length and respondent burden. Therefore, the number of items used to measure a multifaceted behavior, such as information seeking on behalf of others, may not fully capture the constructs of interest. Use of self-report data introduces the possibility of recall bias. Finally, it is important to recognize that lay sources of information may not always transmit medically accurate or guideline-concordant information. This study did not assess health literacy level and did not explore the content or quality of health information or advice transmitted.

### Conclusion

In a national sample, a high proportion of respondents, including those in the lowest income levels and those born outside the US, report acting as brokers of health information. Brokers more frequently engaged in a variety of online behaviors including health information seeking, creation of online content and downloading of health information onto a mobile device. Members of traditionally disadvantaged groups who report acting as health information brokers display these same behaviors and future studies should examine whether and how these brokers are narrowing the existing digital divide. Directing brokers to high-quality Internet-based resources in familiar online venues or to resources designed for downloading may be an effective way to support dissemination of health information.

#### Practice Implications

Health information brokers have important lessons to teach healthcare professionals about the role they play in disseminating health information and advice to their friends and families. These brokers may be effective targets for initiatives aimed at supporting patient engagement and disease management. In addition, self-identification as a person who engages in brokering behavior may be a marker for those well-positioned to assist in health communication campaigns.

## Abbreviations

EHR: Electronic health record
HINTS: Health Information National Trends Survey
PR: Prevalence ratio
